# Seronegative Rheumatoid Arthritis in an Elderly Dialysis Patient With Multiple Comorbidities: A Case Report

**DOI:** 10.7759/cureus.60066

**Published:** 2024-05-10

**Authors:** Miharu Kawanishi, Shinichiro Suyama, Nozomi Nishikura, Chiaki Sano, Ryuichi Ohta

**Affiliations:** 1 Nephrology, Heisei Memorial Hospital, Unnan, JPN; 2 Internal Medicine, Heisei Meorial Hospital, Unnan, JPN; 3 Community Care, Unnan City Hospital, Unnan, JPN; 4 Community Medicine, Shimane University Faculty of Medicine, Izumo, JPN; 5 Communiy Care, Unnan City Hospital, Unnan, JPN

**Keywords:** family medicine, general medicine, prednisolone, diagnostic challenges, autoimmune diseases, 80 and over, hemodialysis patients elderly, seronegative rheumatoid arthritis

## Abstract

The diagnosis of autoimmune diseases in elderly, immunocompromised patients undergoing dialysis poses significant challenges due to the diverse etiology of symptoms such as fever and systemic pain. This case study reports on a 79-year-old man undergoing hemodialysis with a history of multiple comorbidities, including diabetes, heart failure, and pure red cell aplasia. He presented with subacute polyarthritis and fever and was ultimately diagnosed with seronegative rheumatoid arthritis. The case illustrates the complexities of differential diagnosis in this population, emphasizing the need for a systematic approach to distinguish between possible infectious and autoimmune causes. Despite the absence of rheumatoid factor and anti-citrullinated protein antibody, the patient's clinical presentation and response to steroids supported the diagnosis of seronegative rheumatoid arthritis. Treatment with prednisolone resulted in significant improvement in symptoms and quality of life, demonstrating the effectiveness of steroids in managing autoimmune conditions in elderly, high-risk patients. However, such treatment necessitates careful monitoring due to potential adverse effects. This case underlines the importance of considering autoimmune diseases in differential diagnoses and customizing treatment strategies to accommodate the unique needs of elderly, immunocompromised patients on dialysis. Insights from this case contribute to better understanding and management of complex clinical scenarios in similar patient populations.

## Introduction

Diagnosing elevated inflammatory responses accompanied by arthritis in elderly immunocompromised patients presents a complex challenge due to the broad spectrum of differential diagnoses [1]. This complexity is further magnified in patients undergoing blood dialysis as the potential for infection diversifies significantly [2]. Fever in the elderly is predominantly caused by a constellation of conditions, including autoimmune diseases, malignancies, infections, and vasculitis, necessitating a meticulous approach to differential diagnosis [3]. Among the elderly, late-onset rheumatoid arthritis often manifests serologically with negative rheumatoid factor (RF) and anti-citrullinated protein antibody (ACPA), frequently accompanied by fever [4,5]. This necessitates a logical differentiation between infections and vasculitis, highlighting the intricacies of diagnosing such conditions [6].

We report a case involving an elderly patient undergoing hemodialysis with pure red cell aplasia who presented with subacute polyarthritis and fever. The patient was ultimately diagnosed with seronegative rheumatoid arthritis and treated effectively with a preserved quality of life. This case underscores the diagnostic challenges and emphasizes the importance of a thorough and systematic approach to distinguishing between similar clinical presentations, particularly in complex and high-risk patient populations.

## Case presentation

A 79-year-old man, who was originally independent in activities of daily living (ADL), presented to a rural hospital with the chief complaint of fatigue and fever. One week before his admission, he began to feel fatigued. He had developed a mild fever of 37℃ the day before the admission and was emergently transported to the hospital. He had no upper respiratory or abdominal symptoms such as diarrhea or vomiting. There was no history of overseas travel or contact with infected individuals. Upon examination, he showed no abnormal physical findings regarding the causes of fatigue and fever. The laboratory tests showed elevated inflammatory responses (C-reactive protein of 10.9 mg/dL) and severe anemia with a hemoglobin level of 6 g/dL. He was transferred to our hospital for further investigation and continuity of hemodialysis. The patient underwent thrice-weekly maintenance hemodialysis for end-stage renal failure due to diabetic nephropathy for the past six years in our hospital. His other medical history included type 2 diabetes mellitus, hypertension, aplastic anemia, epilepsy, right purulent arthritis, MRSA bacteremia, benign prostatic hyperplasia, chronic heart failure, angina, lumbar spinal canal stenosis, multiple lacunar strokes, left femoral trochanteric fracture, and takotsubo cardiomyopathy. His medications included levetiracetam 500mg daily, prednisolone 3 mg daily, clopidogrel 75mg daily, bisoprolol 2.5mg daily, vonoprazan 10mg daily, and olmesartan 20 mg daily.

Upon admission to our hospital, the patient was alert with a body temperature of 37.4°C, blood pressure of 152/66 mmHg, pulse rate of 66 beats/min, respiratory rate of 20 breaths/min, and oxygen saturation of 99% on room air. Physical examination revealed no abnormalities in the head, neck, chest, or abdomen. The patient had a cuff-type catheter for hemodialysis inserted in his right internal jugular vein, which was clean and without contamination. He did not show any pain in his right lower leg without swelling or warmth. Blood tests showed severe anemia with a hemoglobin level of 5.4 g/dL, white blood cell count of 6,060/µL, and elevated CRP of 9.19 mg/dL (Table 1).

**Table 1:** Initial laboratory data of the patient CRP, C-reactive protein; Ig, immunoglobulin

Parameter	Level	Reference
White blood cells	6.06	3.5–9.1 × 10^3^/μL
Neutrophils	74.0	44.0–72.0%
Lymphocytes	18.2	18.0–59.0%
Hemoglobin	5.4	11.3–15.2 g/dL
Hematocrit	16.9	33.4–44.9%
Mean corpuscular volume	116.6	79.0–100.0 fl
Platelets	16.9	13.0–36.9 × 10^4^/μL
Erythrocyte sedimentation rate	57	2–10 mm/hour
Total protein	6.2	6.5–8.3 g/dL
Albumin	2.4	3.8–5.3 g/dL
Total bilirubin	0.4	0.2–1.2 mg/dL
Aspartate aminotransferase	14	8–38 IU/L
Alanine aminotransferase	9	4–43 IU/L
Lactate dehydrogenase	191	121–245 U/L
Blood urea nitrogen	58.2	8–20 mg/dL
Creatinine	5.86	0.40–1.10 mg/dL
Serum Na	130	135–150 mEq/L
Serum K	5.2	3.5–5.3 mEq/L
Serum Cl	93	98–110 mEq/L
Ferritin	409	14.4–303.7 ng/mL
CRP	9.19	<0.30 mg/dL
IgG	1520	870–1700 mg/dL
IgM	158	35–220 mg/dL
IgA	241	110–410 mg/dL
Urine test	-	-
Leukocyte	100	Negative
Protein	3+	Negative
Blood	2+	Negative

**Figure 1:**
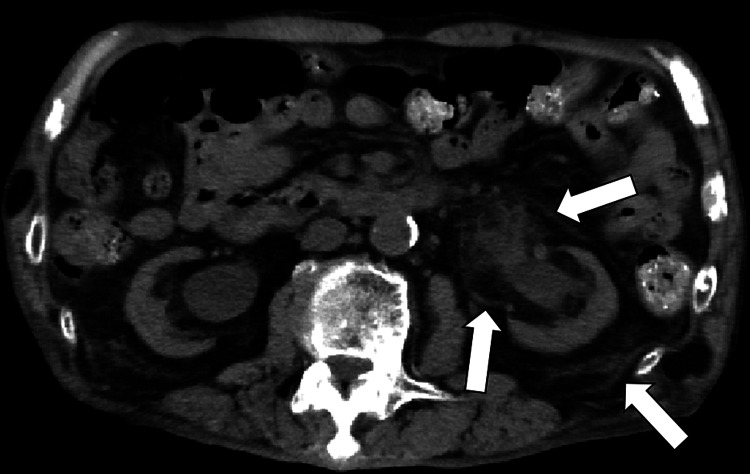
A computed tomography scan of the abdomen showing increased fat tissue density around the left kidney (white arrows)

Urinalysis revealed over 100 white blood cells/HPF and bacteriuria. Two sets of blood cultures were taken, both of which were negative. A computed tomography (CT) scan of the abdomen showed increased fat tissue density around the left kidney, unchanged from before (Figure 1).

**Figure 2:**
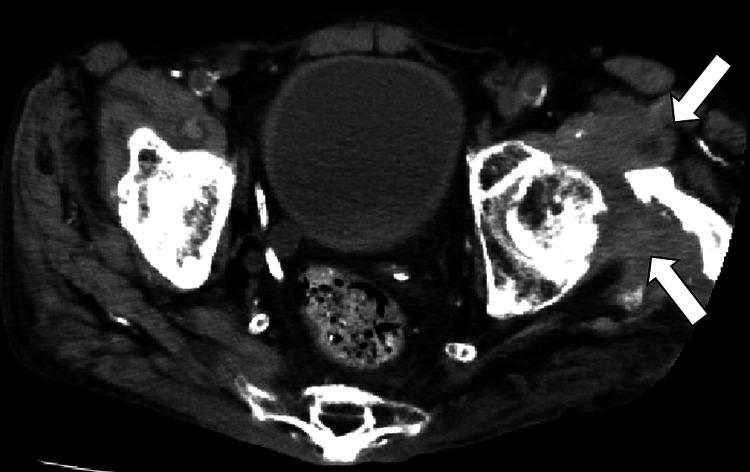
Femoral computed tomography showing multiple masses with internal low absorption areas in the left thigh

There were no physical signs suggesting pneumonia or respiratory disease upon admission. Although pyuria and bacteriuria were present, there was no fever, and considering the patient was undergoing dialysis, it was not conclusively diagnosed as a urinary tract infection. The patient had a cuff-type catheter in place, and while catheter infection was considered, procalcitonin was negative, and two sets of blood cultures were negative, making it unlikely. Multiple masses with internal low absorption areas in the left thigh were seen on CT, prompting suspicion of abscesses, but the puncture yielded no bacteria (Figure 2).

Rapid inflammatory response and anemia progression raised the possibility of antineutrophil cytoplasmic antibodies (ANCA)-associated vasculitis, but tests for myeloperoxidase ANCA and protease 3-ANCA were negative. Anti-nuclear antibodies were also negative. Interferon-Gamma Release Assays (IGRA) were performed to exclude tuberculosis, which was negative. The patient was on long-term steroids, putting him at risk for fungal infections, which were considered in the differential diagnosis. β-D-glucan was positive (46 pg/mL (reference, <20)), and Aspergillus antigen was also positive (>0.5 cut-off index (reference, <0.5)). Still, imaging did not suggest aspergillosis, so it was deemed non-significant, and observation was continued.

Until day 8 of hospitalization, the patient's fever subsided to the 36°C range without additional medication. However, a fever of 38°C appeared on the ninth day of hospitalization. Given the presence of pyuria, bacteriuria, and increased fat tissue density around the left kidney on CT, the patient was diagnosed with urinary tract infection and treated with tazobactam/piperacillin 3.5g/day. The fever was resolved at 36°C the day after starting tazobactam/piperacillin. Urine culture detected methicillin-resistant coagulase-negative staphylococci and Corynebacterium, both sensitive to the ST combination, so the antimicrobial was switched to this combination on the 13th day of hospitalization. However, C-reactive protein persisted high, and low-grade fever continued.

He was discharged home on the 12th day of hospitalization at the patient's request. Still, low-grade fever and general fatigue persisted, leading to a visit to the general medicine department on the 19th day. During the examination, the patient exhibited polyarthritis of bilateral shoulders, wrists, and knees. Based on clinical findings, the possibilities of seronegative rheumatoid arthritis and multiple pseudogout were considered. Given the patient's status as a dialysis patient, treatment with etodolac was initiated for pseudogout. Although the fever subsided, the patient developed a tendency towards drowsiness. Considering the concerns about NSAID-induced consciousness disturbance prompted the discontinuation of the medication. The patient's mental status improved a few days later after the discontinuation. Persistent systemic pain warranted a re-evaluation of physical findings, confirming the persistence of polyarthritis and bursitis. Considering a diagnosis of Seronegative Rheumatoid Arthritis and the patient's hemodialysis status, treatment was initiated with 10 mg of prednisolone (PSL) daily. Subsequently, the inflammatory response decreased, and symptoms gradually improved. Due to the progression of edema and poor blood removal during dialysis, the PSL dose was quickly reduced, resulting in an improvement in the activity of daily life.

## Discussion

In this case, we encountered an elderly patient who was undergoing hemodialysis and had blood disorders, eventually diagnosed with seronegative rheumatoid arthritis. Despite being immunocompromised, symptom remission was achieved using PSL. This case highlights the importance of considering non-infectious diseases in the differential diagnosis of fever and systemic pain in elderly patients, particularly those undergoing dialysis. The persistence of inflammation can deplete a patient's physical strength, suggesting the importance of not hesitating to treat with steroids or similar medications once infections have been ruled out, even in patients prone to infections due to aging, hemodialysis treatment, or other factors.

Elderly patients, especially those in an immunocompromised state, may present with non-specific symptoms like fever and systemic pain due to a variety of causes [7,8]. Patients receiving hemodialysis, as in this case, are already in a compromised immune state, making them more vulnerable to various infections and autoimmune diseases [9]. Such patients require a broad differential diagnosis to identify the cause of fever, which can sometimes take considerable time.

Clinical features of rheumatoid arthritis characterize seronegative rheumatoid arthritis despite the absence of anti-CCP antibodies and rheumatoid factor. In this case, the absence of signs of infection in standard blood tests increased the suspicion of an autoimmune disease. However, the seronegative status can delay diagnosis, posing a significant challenge for clinicians in managing symptoms and choosing treatments [10,11]. Steroids like PSL play a crucial role in treating many autoimmune diseases due to their potent anti-inflammatory effects, as evidenced by symptom remission and improved quality of life in this patient [12]. However, the use of steroid treatment also carries the risk of side effects such as osteoporosis, diabetes, and increased infection risk, necessitating a careful evaluation of the benefits and risks of treatment [13].

This case underscores the complexity of diagnosing fever and systemic pain in elderly or immunocompromised patients and the difficulties in selecting treatments for autoimmune diseases. Customized diagnostic and treatment strategies are required according to the individual patient's situation, especially for elderly patients with unique backgrounds, such as those undergoing hemodialysis [14].

The successful use of steroid treatment was a critical turning point in this case, highlighting the need for careful risk management of long-term side effects in elderly or immunocompromised patients. Minimizing the effective dose and considering the timing for tapering or discontinuing treatment is essential [15]. Moreover, adjunctive therapies and lifestyle adjustments to maintain quality of life while controlling inflammation are crucial [16]. Managing complex cases like elderly or dialysis patients requires interdisciplinary teamwork among physicians, nurses, pharmacists, dietitians, and rehabilitation staff to provide comprehensive care tailored to each patient's needs, ultimately improving treatment outcomes in rural contexts [17].

Finally, unique cases like this provide valuable insights for medical research and are beneficial for the education of future clinicians. As understanding the diagnosis and treatment of autoimmune diseases deepens, more effective treatment methods and management strategies may be developed [18]. Research on disease characteristics and treatment responses in elderly patients or those with specific chronic conditions is essential for improving the quality of healthcare provision [19]. The lessons learned from this case serve as essential guidelines for clinicians in assessing and managing complex symptoms in elderly or immunocompromised patients and contribute to better patient care through clinical education.

## Conclusions

This case report underscores the complexities of diagnosing and treating seronegative rheumatoid arthritis in elderly, immunocompromised dialysis patients with a diverse range of comorbidities. It highlights the importance of a comprehensive differential diagnosis that includes autoimmune diseases in evaluating non-specific symptoms like fever and systemic pain beyond the initial focus on infectious etiologies. The effective management of this patient with prednisolone, despite the inherent risks of steroid therapy in such a vulnerable population, illustrates the critical balance between therapeutic benefit and potential adverse effects. This case contributes valuable insights into personalized care strategies for managing autoimmune diseases in high-risk elderly patients.
